# Assessment of Gluten-Free Products’ Availability and Satisfaction in a Polish Population of Coeliac Disease Patients and Their Caregivers

**DOI:** 10.3390/nu16203460

**Published:** 2024-10-12

**Authors:** Dominika Głąbska, Dominika Guzek, Dominika Skolmowska, Frank Vriesekoop

**Affiliations:** 1 Department of Dietetics, Institute of Human Nutrition Sciences, Warsaw University of Life Sciences (SGGW-WULS), 159C Nowoursynowska Street, 02-776 Warsaw, Poland; dominika_glabska@sggw.edu.pl (D.G.); dominika_skolmowska@sggw.edu.pl (D.S.); 2 Department of Food Market and Consumer Research, Institute of Human Nutrition Sciences, Warsaw University of Life Sciences (SGGW-WULS), 159C Nowoursynowska Street, 02-776 Warsaw, Poland; 3 Harper Food Innovation, Harper Adams University, Newport TF10 8NB, UK; fvriesekoop@harper-adams.ac.uk

**Keywords:** coeliac disease, gluten-free diet, gluten-free products, consumers, satisfaction, availability

## Abstract

Background/Objectives: Among the most important challenges associated with the gluten-free diet are the high costs and limited availability of gluten-free products, accompanied by the lower nutritional value of gluten-free products. The aim of the presented study was to assess gluten-free products’ availability and satisfaction in a Polish population of coeliac-disease patients and their caregivers. Methods: The study was conducted in a population of Polish female coeliac-disease patients and female family members/relatives of patients diagnosed with coeliac disease, being members of the Polish Coeliac Society and purchasing gluten-free products. A population of *n* = 819 was included in the studied group based on the inclusion and exclusion criteria (*n* = 547 of patients and *n* = 272 of family members/relatives of patients). The study was conducted as a part of an international project to assess the opinions of coeliac-disease patients about gluten-free products, as well as the availability and prices of gluten-free products in various countries, while an identical questionnaire was applied in all participating countries, with transcultural adaptation applied. Opinions concerning the availability of and satisfaction with gluten-free products were assessed based on a questionnaire of agreement with fixed statements about the accessibility, range and quality of gluten-free products in Poland, with a five-point Likert scale to declare the agreement. This was stratified by the following variables: age, place of residence, being diagnosed with coeliac disease, place of purchasing major grocery shopping, gluten-free products at least occasionally bought online, declared problem(s) with the availability and quality of gluten-free products. Results: While comparing the studied sub-groups, it may be stated that some of them were more satisfied than the other sub-groups with the gluten-free products, including their availability and quality; namely, older respondents were more satisfied than younger ones (*p* < 0.05), respondents living in small towns/villages were more satisfied than those living in big cities (*p* < 0.05), respondents undertaking major grocery shopping in hypermarkets were more satisfied than those not doing this (*p* < 0.05), and respondents not buying gluten-free products online were more satisfied than those undertaking this at least occasionally (*p* < 0.05). At the same time, respondents diagnosed with coeliac disease were more satisfied with the availability and less satisfied with the quality of gluten-free products, while respondents with diagnosed family members/relatives were less satisfied with the availability and more satisfied with the quality (*p* < 0.05). Conclusions: The group of female coeliac-disease patients and female family members/relatives of patients diagnosed with coeliac disease was highly diverse in terms of their satisfaction with gluten-free products’ availability and quality, whilst older respondents, respondents living in small towns/villages, respondents doing major grocery shopping in hypermarkets, and respondents not buying gluten-free products online were more satisfied. Respondents with family members/relatives diagnosed with coeliac disease declared serious efforts and sacrifice to purchase gluten-free products, which was associated with their higher satisfaction with quality and lower satisfaction with availability, while respondents diagnosed with coeliac disease chose easier options, resulting in their higher satisfaction with availability and lower satisfaction with quality.

## 1. Introduction

Coeliac disease (also called coeliac sprue or gluten-sensitive enteropathy) is defined as a chronic autoimmune disease affecting the small intestine in genetically predisposed individuals and is associated with an inappropriate immune response to the intake of gluten, causing small intestinal inflammation and damage [1], and it is clinically classified as classic, non-classic, subclinical, potential, and refractory [2]. Based on a recent systematic review and meta-analysis by Singh et al. [3], it may be indicated that the prevalence of coeliac disease is estimated as 1.4% (diagnosed based on serologic results) or 0.7% (diagnosed based on biopsy results), with a higher prevalence for children than adults and higher prevalence for female individuals than for male ones. Diet therapy for coeliac disease is the only possible treatment [4], which leads to a reduction in disease symptoms, prevents the risk of disease complications, and improves the quality of life [5]. However, potential novel management therapies of coeliac disease are currently being studied, including polymerizing binders, anti-zonulin therapy, tissue transglutaminase inhibitors, silencing RNA therapies, enzyme therapies, DQ2/DQ8 inhibitors, monoclonal antibodies, coeliac vaccines, and stem cell therapy [6].

Diet therapy for coeliac disease is based on the life-long following of the gluten-free diet, defined as complete avoidance of both gluten-containing food products (such as wheat, rye, spelt, and barley) and those that may be cross-contaminated with gluten, in order to reduce total daily gluten supply to a very low level—as recommended by the British Society of Gastroenterology, to a level below 10 mg gluten per day [7]. In order to reduce the gluten supply, gluten-free products with confirmed low gluten content are necessary [8], which is supported by the European Commission regulation, indicating that a product may be referred to as ‘gluten-free’ only if it contains less than 20 mg of gluten per 1 kg of food product (20 ppm of gluten) [9].

It is suggested that strict adherence to the gluten avoidance recommendation is not observed in all coeliac disease-diagnosed patients and depends on various factors, including ethnicity and age at diagnosis, but also on the definition of a gluten-free diet and method of its assessment, which results in from 42 to 91% of patients being defined as strictly following the recommended gluten-free diet [10]. Among the most important challenges associated with the gluten-free diet, indicated by patients following it, are the high cost of gluten-free products, the limited availability of gluten-free products in markets, and the limited availability of gluten-free meals in restaurants [11]. Without a wide range of gluten-free products in markets and a wide range of gluten-free meals in restaurants available for all patients for a reasonable price, following a gluten-free diet may result in nutritional deficiencies but also in social and psychological problems and barriers [12]. 

On the one hand, the price is, for some patients, a serious problem, which is reflected by the analysis conducted for the United States of America, revealing that gluten-free products are 183% more expensive than their gluten-containing counterparts, while such difference in prices is observed for all regions and venues [13]. On the other hand, even if some patients are motivated to follow a gluten-free diet, independently of its price, the availability of gluten-free products is very limited, which is reflected by the analysis conducted for Great Britain based on data gathered for almost 10 years, revealing that the availability of gluten-free products is increasing slowly and mainly for premium markets (not for budget and convenience ones), where they are even more expensive [14]. This corresponds with the results of a Polish study revealing that for coeliac-disease patients, their economic situation is among the most important determinants of their quality of life, while for other factors that are also supposed to be important (including disease duration or gluten-free diet adherence), there was no significant association [15]. 

Last but not least, there is a problem with the quality of gluten-free products, which are commonly of lower nutritional value and may result in the development of diet-related diseases due to a higher content of saturated fat, simple carbohydrates and salt, accompanied by lower contents of protein and fibre [16]. At the same time, coeliac-disease patients are often confused by the quality of gluten-free products due to there being many additional features of gluten-free products communicated on the packaging [17]. Its consequence is a relatively high food neophobia level in coeliac disease individuals, which means that they prefer well-known food products to novel ones, even if they are also gluten-free [18].

Taking into account the described challenges associated with gluten-free products, the aim of the presented study was to assess the gluten-free products’ availability and satisfaction in a Polish population of coeliac-disease patients and their caregivers. As in a lot of countries, including Poland [19], mainly women are responsible for meal preparation and grocery purchasing in families [20]; this study was planned to be conducted in a population of female respondents only, which was a common approach also for the previous studies analysing the gluten-free products choices in a Polish population [15]. This corresponds a higher incidence of coeliac disease in women than in men [21], as well as the fact that women are more focused on their health than men [22]. The study was conducted as a part of an international project to assess the opinions of coeliac-disease patients about gluten-free products, as well as the availability and prices of gluten-free products in various countries [23,24], which was developed in order to study the general situation of coeliac patients.

## 2. Materials and Methods

### 2.1. Ethical Background

The presented part of the project was conducted in cooperation of the Institute of Human Nutrition Sciences, Warsaw University of Life Sciences (SGGW-WULS) in Poland (responsible for data gathering and analysis), Polish Coeliac Society (responsible for questionnaire dissemination and contact with Society members), and Harper Adams University in the United Kingdom (responsible for project administration and coordination). 

This study was conducted in a period from May to August 2022 based on the computer-assisted web interview (CAWI) method, and the project details were presented in a previous publication [23]. This study was conducted based on ethical approval obtained within Harper Adams University, United Kingdom (No. 0439-202106-STAFF-CO2). All the procedures were in agreement with the Declaration of Helsinki. All participants of the study provided informed consent for study participation.

### 2.2. Study Population

The population presented within the study was recruited by the Polish Coeliac Society, which is a member of the Association of European Coeliac Societies (AOECS) and participates in scientific research of coeliac-disease patients conducted in Poland [25], from its members, while the inclusion criteria were as follows:

- Being a member of the Polish Coeliac Society (members with coeliac disease diagnosed and those with family members diagnosed with coeliac disease allowed);
- Informed consent to study participation.

The exclusion criteria were as follows:

- Male gender;
- Age < 18 years;
- Not living in Poland;
- Not purchasing in Poland gluten-free products;
- Any missing data in the completed questionnaire.

The questionnaire was completed by *n* = 978 respondents, but due to exclusion criteria, *n* = 159 of them were excluded from the analysis, as they were male (*n* = 95), under the age of 18 years (*n* = 36), not living in Poland (*n* = 2), or not purchasing gluten-free products in Poland (*n* = 26). The final sample size in the analysed sample was *n* = 819. Within the exclusion criteria, it was not indicated that patients and their family members could not both participate in the study if they were both adults and purchased gluten-free products in Poland, as each of them presented their own perspective.

### 2.3. Questionnaire

The study was based on the universal questionnaire developed to assess the opinions of coeliac-disease patients about gluten-free products in the international population, as presented in the previous study [23]. The study aimed to assess the following issues: purchasing habits, place of purchasing gluten-free products and other food products, type of purchased gluten-free products and purchasing frequency, availability of gluten-free products, purchasing gluten-free products online and any other activity to obtain gluten-free products, quality of available gluten-free products and their attributes, interpretation of gluten-free labels, and gaps in the gluten-free product market, all in the context of socio-demographic characteristics of respondents.

In the presented analysis, opinions concerning the availability of and satisfaction with gluten-free products were the major results. They were assessed based on a questionnaire of agreement with fixed statements about the accessibility, range and quality of gluten-free products in Poland with a 5-point Likert scale to declare their agreement (scale from 1 = strongly disagree to 5 = strongly agree), as described for Australia and New Zealand [23]. The 5-point Likert scale was chosen as it allowed us to obtain high-quality data [26,27]. The applied fixed statements list was as follows:

- I am satisfied with the range of gluten-free products available in the store/market;
- I am satisfied with the quality of gluten-free products available in the store/market;
- I believe that the gluten-free products available in the store/supermarket are as good as their gluten-containing counterparts;
- I like gluten-free products that I buy in a store/supermarket;
- I trust the information on the labels of products marked as gluten-free in a store/market;
- I prefer to make my own gluten-free products whenever possible;
- I believe that when I buy gluten-free products in a store/supermarket, I get good quality at a reasonable price;
- I often have to go to several different stores to get the gluten-free products I need;
- Sometimes I have to go to another city to buy the gluten-free products I need;
- I think the shelves in the “free” section of the store/supermarket are well-stocked.

The participants were also asked about their basic characteristics, including their socio-demographic characteristics, the issues necessary to verify the exclusion criteria, and those included to analysis as variables, as follows:

- Gender (closed-ended single-choice question with a choice of female, male, other and refused to answer);
- Age (closed-ended single-choice question with a choice of age categories as follows: <18, 18–24, 25–34, 35–44, 45–54, 55–64, 65–74, 75–84, and >84 years);
- Place of residence, in order to verify living in Poland and to stratify respondents by place of residence (open-ended question about the zip code);
- Being diagnosed with coeliac disease or having a family member/relative diagnosed with coeliac disease (closed-ended single-choice question about the reason for purchasing gluten-free products, with a choice of being diagnosed with coeliac disease, having a family member/relative diagnosed with coeliac disease and other reasons, with an additional request to specify the other reason if this option chosen);
- Place of purchasing major grocery shopping, in order to verify purchasing gluten-free products in Poland and to stratify respondents by purchasing in hypermarkets and not purchasing (open-ended question about the names of the stores/markets);
- Purchasing gluten-free products online at least occasionally (closed-ended single-choice question with a choice of yes and no categories);
- Declared problem with the availability of gluten-free products (closed-ended single-choice question with a choice of yes and no categories);
- Declared problem with the quality of gluten-free products (closed-ended single-choice question with a choice of yes and no categories).

Due to the fact that the study was conducted as a part of an international project, and an identical questionnaire was applied in all participating countries, the original questionnaire was developed in English and was translated, to be used in all participating countries, into national languages, while transcultural adaptation was also applied. On the basis of the guidelines of the World Health Organization (WHO) [28], and in agreement with a commonly applied procedure [29], the process of translation with transcultural adaptation included forward translation from English to Polish, backward translation from Polish to English, and the final polishing of the questionnaire in order to ensure conceptual, semantic, idiomatic, and cultural equivalence.

### 2.4. Data Analysis

In the presented analysis, the availability of and satisfaction with gluten-free products, based on a questionnaire of agreement with a 5-point Likert scale, were stratified by the following variables:

- Age (information based on the answer to a simple question about age)—stratified into 2 sub-groups: age ≤ 44 years (*n* = 662) and age ≥ 45 years (*n* = 157). The age range was based on the categories of early adulthood/early middle age (age ≤ 44 years) and late middle age/late adulthood (age ≥ 45 years), in agreement with the categories indicated by other authors [30].
- Place of residence (information based on the answer to a question about zip code)—stratified into 2 sub-groups: living in a big city (*n* = 400) and living in a small town/village (*n* = 419). A big city was defined as city of over 100,000 inhabitants, based on the definition by the Central Statistical Office in Poland (VI and VII class of the size of cities [31]) and in agreement with the categories indicated by other authors [32].
- Being diagnosed with coeliac disease (information based on the answer to a simple question about it)—stratified into 2 sub-groups: diagnosed with coeliac disease (*n* = 547) and family member/relative diagnosed with coeliac disease (*n* = 272). This was because it is indicated that purchasing gluten-free products and preparing gluten-free meals are challenging not only for coeliac-disease patients but also their family members [33].
- Place of purchasing major grocery shopping (information based on the answer to a question about the name of their chosen stores/markets)—stratified into 2 sub-groups: major grocery shopping in hypermarkets (*n* = 595) and major grocery shopping not in hypermarkets (*n* = 224). A hypermarket was defined as a market of over 2500 m^2^, based on the definition by the Central Statistical Office in Poland [34], and the size of markets was verified based on the trade press market analysis [35].
- Gluten-free products at least occasionally bought online (information based on the answer to a simple question about it)—stratified into 2 sub-groups: gluten-free products at least occasionally bought online (*n* = 699) and gluten-free products not bought online (*n* = 120).
- Declared problem with the availability of gluten-free products (information based on the answer to a simple question about it)—stratified into 2 sub-groups: declared problem with the availability of gluten-free products (*n* = 795) and no declared problem with the availability of gluten-free products (*n* = 24).
- Declared problem with the quality of gluten-free products (information based on the answer to a simple question about it)—stratified into two sub-groups: declared problem with the quality of gluten-free products (*n* = 509) and no declared problem with the quality of gluten-free products (*n* = 310).

### 2.5. Statistical Analysis

To verify the distribution of the data, the Shapiro–Wilk test was used. Based on nonparametric distribution, the Mann–Whitney U test was used to compare sub-groups.

A level of *p* ≤ 0.05 was treated as a significant difference between sub-groups. Statistical analysis was conducted using Statistica 8.0 (Statsoft Inc., Tulsa, OK, USA).

## 3. Results

Table 1 presents the characteristics and demographics of the study participants (*n* = 819). The majority of respondents were aged 35–44 years (44%), lived in small towns or villages (51%), were diagnosed with coeliac disease (67%), declared major grocery shopping in hypermarkets (73%), purchased gluten-free products online at least occasionally (85%), and declared problems with gluten-free product availability (97%) and quality (62%).

Table 2 presents opinions about gluten-free products’ availability and satisfaction in the studied group of Polish female coeliac-disease patients and female family members/relatives of patients diagnosed with coeliac disease, stratified by age. Compared with younger respondents, older respondents were more satisfied with the quality of gluten-free products available in stores/markets (*p* = 0.0061), declared that they believe more that the gluten-free products available in stores/supermarkets are as good as their gluten-containing counterparts (*p* = 0.0003), and declared that they believe more that when they buy gluten-free products in a store/supermarket, they receive good quality at a reasonable price (*p* = 0.0214). At the same time, older respondents, compared with younger ones, agreed more with the declaration that they prefer to make their own gluten-free products whenever possible (*p* = 0.0024), but they agreed less with the declaration that they have to go to several different stores to buy the gluten-free products they need (*p* = 0.0183). 

Table 3 presents opinions about gluten-free products’ availability and satisfaction in the studied group of Polish female coeliac-disease patients and female family members/relatives of patients diagnosed with coeliac disease, stratified by place of residence. Compared with respondents living in big cities, those living in small towns/villages declared that they believe more that the gluten-free products available in stores/supermarkets are as good as their gluten-containing counterparts (*p* = 0.0057), and declared that they believe more that when they buy gluten-free products in a store/supermarket, they receive good quality at a reasonable price (*p* = 0.0446). At the same time, respondents living in small towns/villages, compared with those living in big cities, agreed more with the declaration that they prefer to make their own gluten-free products whenever possible (*p* = 0.0242), and they agreed more with the declaration that they sometimes have to travel to another city to buy the gluten-free products they need (*p* < 0.0001).

Table 4 presents opinions about gluten-free product availability and satisfaction in the studied group of Polish female coeliac-disease patients and female family members/relatives of patients diagnosed with coeliac disease, stratified by the diagnosis of coeliac disease. Compared with respondents diagnosed with coeliac disease, those with family members/relatives diagnosed with coeliac disease were less satisfied with the range of gluten-free products available in stores/markets (*p* = 0.0001) but declared that they believe more that the gluten-free products available in stores/supermarkets are as good as their gluten-containing counterparts (*p* = 0.0264). At the same time, respondents with family members/relatives diagnosed with coeliac disease, compared with those diagnosed with coeliac disease, agreed more with the declaration that they prefer to make their own gluten-free products whenever possible (*p* = 0.0444), that they have to go to several different stores to buy the gluten-free products they need (*p* = 0.0097), and that they sometimes have to travel to another city to buy the gluten-free products they need (*p* = 0.0259).

Table 5 presents opinions about gluten-free products’ availability and satisfaction in the studied group of Polish female coeliac-disease patients and female family members/relatives of patients diagnosed with coeliac disease, stratified by primary place of purchasing major grocery shopping. While compared with respondents not completing their primary grocery shopping in hypermarkets, those who did so declared that they were more satisfied with the range (*p* = 0.0150) and quality of gluten-free products available in stores/markets (*p* = 0.0223) and agreed less with the declaration that they sometimes have to travel to another city to buy the gluten-free products they need (*p* = 0.0114).

Table 6 presents opinions about gluten-free products’ availability and satisfaction in the studied group of Polish female coeliac-disease patients and female family members/relatives of patients diagnosed with coeliac disease, stratified by whether gluten-free products were at least occasionally bought online. Compared with respondents buying gluten-free products online at least occasionally, those not buying them online declared that they are more satisfied with the range (*p* = 0.0253) and quality of gluten-free products available in stores/markets (*p* = 0.0316). At the same time, respondents buying gluten-free products online at least occasionally, compared with those not buying them online, agreed more with the declaration that they prefer to make their own gluten-free products whenever possible (*p* = 0.0430), that they often have to go to several different stores to buy the gluten-free products they need (*p* = 0.0082), and that they sometimes have to travel to another city to buy the gluten-free products they need (*p* = 0.0001).

Table 7 presents opinions about gluten-free products’ availability and satisfaction in the studied group of Polish female coeliac-disease patients and female family members/relatives of patients diagnosed with coeliac disease, stratified by a declared problem with the availability of gluten-free products. Compared with respondents stating a problem with gluten-free products’ availability, those not stating this declared that they were more satisfied with the range of gluten-free products available in stores/markets (*p* = 0.0008), believe more that the gluten-free products available in the store/supermarket are as good as their gluten-containing counterparts (*p* = 0.0245), declared that they like more gluten-free products that they buy in a store/supermarket (*p* = 0.0093), and declared more strongly that they perceive the shelves in the “free” section of the store/supermarket to be well stocked (*p* = 0.0009). At the same time, respondents stating a problem with gluten-free product availability, compared with those not stating this, agreed more with the declaration that they often have to go to several different stores to buy the gluten-free products they need (*p* = 0.0001), and that they sometimes have to travel to another city to buy the gluten-free products they need (*p* = 0.0017).

Table 8 presents opinions about gluten-free products’ availability and satisfaction in the studied group of Polish female coeliac-disease patients and female family members/relatives of patients diagnosed with coeliac disease, stratified by declared problem with the quality of gluten-free products. Compared with respondents stating a problem with gluten-free product quality, those not stating this declared that they are more satisfied with the range (*p* = 0.0282) and quality of gluten-free products available in stores/markets (*p* < 0.0001), believe more that the gluten-free products available in the store/supermarket are as good as their gluten-containing counterparts (*p* < 0.0001), declared that they like more gluten-free products that they buy in stores/supermarkets (*p* < 0.0001), that they trust the information on the labels of products marked as gluten-free in stores/markets more (*p* = 0.0189), believe more that when they buy gluten-free products in a store/supermarket, they receive good quality at a reasonable price (*p* < 0.0001), and declared more strongly that they perceive the shelves in the “free” section of stores/supermarkets to be well stocked (*p* < 0.0001).

## 4. Discussion

Analysis of the declared agreement with the statements in the sub-groups stratified based on a declared problem with the availability and quality of gluten-free products confirmed that the applied questionnaire of level of agreement with fixed statements about the accessibility, range and quality of gluten-free products in Poland, with a five-point Likert scale to declare the level of agreement, is a good tool to assess the opinions of the studied group. This analysis confirmed a high agreement—respondents defining themselves as having problems with gluten-free products’ availability and quality were revealed as being less satisfied in the questionnaire. At the same time, the applied questionnaire provided more detailed information, allowing specific problems to be defined—an inadequate range of gluten-free products, shelves in the “free” section not being well-stocked, gluten-free products not being as good as their gluten-containing counterparts, not trusting the information on the labels of products marked as gluten-free, and not feeling that the buyer of gluten-free products receives good quality at a reasonable price.

The other authors indicate that the problem with gluten-free products is more complex—inadequate availability and quality, accompanied by high prices on the one hand, but inadequate knowledge of gluten-free products by consumers on the other hand [36]. This lack of knowledge may be a crucial issue, as it may be supposed that coeliac-disease patients with inadequate knowledge may not understand the technology of gluten-free product manufacturing, so they may be disappointed with the availability and quality, which are commonly, in their opinion, insufficient [37]. Interesting observations were formulated in a study by Pohoreski et al. [38], in which 63% of the studied coeliac disease adolescents had insufficient knowledge about their disease, and within this sub-group, 56% were not able to properly identify gluten-free products, while at the same time, 88% of the studied group assessed their gluten-free diet adherence as good. The other issue is associated with the fact that coeliac-disease patients have relatively low general nutritional knowledge, being lower than for patients with other gastrointestinal diseases and even lower than in healthy subjects, which results from the fact that they are focused solely on gluten avoidance and believe that diet based on gluten-free products is healthy for them and no other actions are needed [39]. It is associated with some false beliefs, currently becoming common in groups of consumers with a lack of adequate nutritional knowledge, that gluten-free products are for the general population the best option to choose, due to the fact that they may promote not only better health, but also body mass reduction [40] or physical performance [41]. A similar situation was reflected in the study by Sangiorgio et al. [42] for a sample of gluten-free product consumers, indicating that commonly, not only coeliac-disease patients and other patients with gluten-related diseases purchase gluten-free products, but also healthy individuals who perceive them as healthier, or believe that consuming them may allow them not to develop coeliac disease, in spite of the fact that they do not understand what coeliac disease is and how gluten-free products are manufactured. As a result, some market analyses indicate that coeliac-disease patients are not the most important group of consumers of gluten-free products, but healthy individuals [43]. This is a specific group of consumers who are health-conscious and weight-conscious but not educated and who blame gluten for all possible health problems [44], which means that they follow a gluten-free diet that is not better for them than a regular diet [45], or is even unsuitable [46,47], but makes them the driving force for the gluten-free product market [48].

Comparing the studied sub-groups, it may be stated that some of them were more satisfied than other sub-groups with gluten-free products, including their availability and quality; namely, older respondents were more satisfied than younger ones, respondents living in small towns/villages were more satisfied than those living in big cities, respondents completing major grocery shopping in hypermarkets were more satisfied than those not doing this, and respondents not buying gluten-free products online were more satisfied than those doing this at least occasionally. However, for the diagnosis of coeliac disease, there was an interesting difference, as diagnosed respondents were more satisfied with availability and less satisfied with quality, while respondents with diagnosed family members/relatives were less satisfied with availability and more satisfied with quality. Some of the observed associations are easy to explain, while others seem to be more complex. 

Older respondents were more satisfied with gluten-free products’ availability and quality than younger respondents, which may be a result of the constantly growing gluten-free product market, as according to Statista [49], in 2022, the global gluten-free product market was valued at USD 6.7 billion, in 2024—USD 7.74 billion, and in 2032, it is expected to be valued at USD 14 billion, so it may be supposed that the respondents had observed this growth. Respondents completing major grocery shopping in hypermarkets were more satisfied with gluten-free products’ availability and quality than those not doing this, which may be a result of the better choice and lower prices of gluten-free products in supermarkets than local grocery and corner shops [50]. Respondents not buying gluten-free products online were more satisfied with gluten-free products’ availability and quality than those doing so at least occasionally, as it may be supposed that if they are less satisfied, they try to find satisfying products, e.g., by purchasing them online, which is associated with increasing online gluten-free product availability [14].

However, it may be surprising that respondents living in small towns/villages are more satisfied with gluten-free products’ quality than those living in big cities. In general, it may be expected that in big cities, gluten-free products’ availability and quality are better due to the range of shops, their size and variety, which may be confirmed with the general observation that diet adherence in coeliac-disease patients from rural areas is worse than for those from urban areas [51]. However, another study revealed that coeliac-disease patients and family members/relatives of patients diagnosed with coeliac disease reported a similar level of difficulty in finding gluten-free products, independently of living in rural or urban areas [52]. The presented study revealed even more surprising results, as rural respondents not only did not declare a lower level of satisfaction with gluten-free products’ quality, but they declared it higher. This may be explained by another observation, as respondents living in small towns/villages, compared with those living in big cities, more often confirmed that they sometimes have to travel to another city to buy the gluten-free products they need, which may indicate that they are satisfied with the products that they purchase, but they do not purchase it in their hometown. This is probably influenced by the general association that greater effort to gain products influences higher satisfaction with the products, independently of their objective quality features [53], which may cause higher satisfaction with gluten-free products purchased in another city with greater effort.

The other interesting difference was associated with the difference between respondents diagnosed with coeliac disease and those with diagnosed family members/relatives, as diagnosed respondents were more satisfied with the availability and less satisfied with the quality of gluten-free products, while respondents with diagnosed family members/relatives were less satisfied with the availability and more satisfied with the quality. This may be a result of the general approach of women in families—making more effort for other family members, especially children, than for themselves [54], which results from the social expectation that a “good mother” should do so [55], especially for children with chronic diseases [56], and resulting in the perception of motherhood as a sacrifice [57]. In order to explain the abovementioned difference between respondents diagnosed with coeliac disease and those with diagnosed family members/relatives, it must be mentioned that those with family members/relatives diagnosed with coeliac disease more often declared that they prefer to make their own gluten-free products whenever possible, that they have to go to several different stores to buy the gluten-free products they need, and that they sometimes have to travel to another city to buy the gluten-free products they need; namely, they declared serious efforts and sacrifice to obtain gluten-free products. Those women were less satisfied with the availability and more satisfied with the quality of gluten-free products, which is now easy to explain—they are not satisfied with the availability, as serious effort is required of them to obtain such products, but they are satisfied with the quality, as their efforts were probably associated with searching for products of the highest quality. At the same time, respondents diagnosed with coeliac disease were more satisfied with availability and less satisfied with quality, as they did not make such an effort, so obtaining gluten-free products was easier for them, but they did not make effort to obtain products that they perceived as the best possible option, so the quality in their opinion was not so high.

The observations formulated within the conducted study may be important for patients, as well as healthcare providers. The indicated sub-populations of coeliac-disease patients and family members/relatives of patients diagnosed with coeliac disease of the lowest satisfaction with gluten-free products’ availability and quality should be educated by their healthcare providers about the quality of gluten-free products and related technological challenges. Moreover, they should be perceived as a special target group by gluten-free product producers, who should study their expectations, which, so far, have not been met, as well as to ensure the adequate availability of gluten-free products. The indicated actions should allow improvements in the availability and quality of gluten-free products for coeliac-disease patients.

In spite of the fact that the presented study provides novel observations about satisfaction with gluten-free products’ availability and quality in a population of female coeliac-disease patients and female family members/relatives of patients diagnosed with coeliac disease, some limitations of the study must be also listed. As the studied group included only female respondents, it was possible to observe a homogenic group of specific characteristics, but this may be perceived as a limitation of the study, as the study does not provide information about male coeliac-disease patients and male family members/relatives of patients diagnosed with coeliac disease. Moreover, only a limited number of variables were studied; the time since diagnosis and family income were not included. Last but not least, it was possible to gather responses from female coeliac-disease patients and their female family members/relatives, but this was not verified within the questionnaire if they are from the same families.

## 5. Conclusions

The group of female coeliac-disease patients and female family members/relatives of patients diagnosed with coeliac disease was highly diverse in terms of their satisfaction with gluten-free products’ availability and quality, whilst older respondents, respondents living in small towns/villages, respondents completing major grocery shopping in hypermarkets, and respondents not buying gluten-free products online were more satisfied. Respondents with family members/relatives diagnosed with coeliac disease declared serious efforts and sacrifice to obtain gluten-free products, which was associated with a higher satisfaction with quality and lower satisfaction with availability, while respondents diagnosed with coeliac disease chose easier options, resulting in a higher satisfaction with availability and lower satisfaction with quality.

## Figures and Tables

**Table 1:** Characteristics and demographics of the study participants.

Variable	Categories	*n* (%)
Age (years)	18–24	53 (6.5%)
	25–34	249 (30.4%)
	35–44	360 (44.0%)
	45–54	128 (15.6%)
	55–64	24 (2.9%)
	65–74	4 (0.5%)
	75–84	1 (0.1%)
Place of residence	Big city (>100,000 inhabitants)	400 (48.8%)
	Small town/village (<100,000 inhabitants)	419 (51.2%)
Coeliac disease	Respondent diagnosed	547 (66.8%)
	Family member/relative diagnosed	272 (33.2%)
Major grocery shopping	Hypermarkets	595 (72.6%)
	Other	224 (27.4%)
Gluten-free products purchased online	At least occasionally	699 (85.3%)
	Never	120 (14.7%)
Problem with gluten-free product availability	Declared	795 (97.1%)
	Not declared	24 (2.9%)
Problem with gluten-free product quality	Declared	509 (62.1%)
	Not declared	310 (37.9%)

**Table 2:** Opinions about gluten-free products’ availability and satisfaction in the studied group of Polish female coeliac-disease patients and female family members/relatives of patients diagnosed with coeliac disease, stratified by age.

Statements about Gluten-Free Products Available in Stores/Markets	Declared Agreement with the Statements * (*n* = 819)				*p*
	Age ≤ 44 Years (*n* = 662)		Age ≥ 45 Years (*n* = 157)		
	Mean ± SD	Median (IQR) **	Mean ± SD	Median (IQR) **	
I am satisfied with the range of gluten-free products available in the store/market	2.58 ± 1.01	3 (1)	2.61 ± 0.98	3 (1)	0.5870
I am satisfied with the quality of gluten-free products available in the store/market	2.86 ± 1.05	3 (2)	3.10 ± 0.94	3 (1)	0.0061
I believe that the gluten-free products available in the store/supermarket are as good as their gluten-containing counterparts	2.58 ± 1.17	2 (1)	2.96 ± 1.21	3 (2)	0.0003
I like gluten-free products that I buy in a store/supermarket	3.24 ± 0.98	3 (1)	3.36 ± 1.03	3 (1)	0.1013
I trust the information on the labels of products marked as gluten-free in a store/market	3.71 ± 1.09	4 (2)	3.62 ± 1.11	4 (1)	0.3372
I prefer to make my own gluten-free products whenever possible	3.55 ± 1.31	4 (2)	3.88 ± 1.29	4 (2)	0.0024
I believe that when I buy gluten-free products in a store/supermarket, I get good quality at a reasonable price	2.36 ± 1.06	2 (1)	2.54 ± 0.99	3 (1)	0.0214
I often have to go to several different stores to get the gluten-free products I need	4.60 ± 0.88	5 (0)	4.50 ± 0.86	5 (1)	0.0183
Sometimes I have to go to another city to buy the gluten-free products I need	2.94 ± 1.74	3 (4)	2.80 ± 1.69	3 (4)	0.3688
I think the shelves in the “free” section of the store/supermarket are well-stocked	2.19 ± 0.97	2 (2)	2.34 ± 0.96	2 (1)	0.0634

* scale from 1 = strongly disagree to 5 = strongly agree; ** nonparametric distribution for all statements (Shapiro–Wilk test; *p* ≤ 0.05); SD—standard deviation; IQR—interquartile range.

**Table 3:** Opinions about gluten-free products’ availability and satisfaction in the studied group of Polish female coeliac-disease patients and female family members/relatives of patients diagnosed with coeliac disease, stratified by place of residence.

Statements about Gluten-Free Products Available in Stores/Markets	Declared Agreement with the Statements * (*n* = 819)				*p*
	Living in a Big City (*n* = 400)		Living in a Small Town/Village (*n* = 419)		
	Mean ± SD	Median (IQR) **	Mean ± SD	Median (IQR) **	
I am satisfied with the range of gluten-free products available in the store/market	2.58 ± 0.99	3 (1)	2.58 ± 1.02	3 (1)	0.9616
I am satisfied with the quality of gluten-free products available in the store/market	2.86 ± 1.05	3 (2)	2.95 ± 1.02	3 (2)	0.2577
I believe that the gluten-free products available in the store/supermarket are as good as their gluten-containing counterparts	2.53 ± 1.16	2 (1)	2.76 ± 1.2	3 (2)	0.0057
I like gluten-free products that I buy in a store/supermarket	3.2 ± 0.94	3 (1)	3.32 ± 1.04	3 (1)	0.0790
I trust the information on the labels of products marked as gluten-free in a store/market	3.71 ± 1.07	4 (2)	3.68 ± 1.12	4 (2)	0.8114
I prefer to make my own gluten-free products whenever possible	3.51 ± 1.34	4 (3)	3.72 ± 1.28	4 (2)	0.0242
I believe that when I buy gluten-free products in a store/supermarket, I get good quality at a reasonable price	2.31 ± 1.02	2 (2)	2.47 ± 1.08	2 (1)	0.0446
I often have to go to several different stores to get the gluten-free products I need	4.55 ± 0.9	5 (1)	4.61 ± 0.86	5 (0)	0.1561
Sometimes I have to go to another city to buy the gluten-free products I need	2.08 ± 1.45	1 (2)	3.71 ± 1.59	5 (3)	<0.0001
I think the shelves in the “free” section of the store/supermarket are well-stocked	2.21 ± 0.95	2 (2)	2.23 ± 1	2 (2)	0.8030

* scale from 1 = strongly disagree to 5 = strongly agree; ** nonparametric distribution for all statements (Shapiro–Wilk test; *p* ≤ 0.05); SD—standard deviation; IQR—interquartile range.

**Table 4:** Opinions about gluten-free products’ availability and satisfaction in the studied group of Polish female coeliac-disease patients and female family members/relatives of patients diagnosed with coeliac disease, stratified by the diagnosis of coeliac disease.

Statements about Gluten-Free Products Available in Stores/Markets	Declared Agreement with the Statements * (*n* = 819)				*p*
	Diagnosed with Coeliac Disease (*n* = 547)		Family Member/Relative Diagnosed with Coeliac Disease (*n* = 272)		
	Mean ± SD	Median (IQR) **	Mean ± SD	Median (IQR) **	
I am satisfied with the range of gluten-free products available in the store/market	2.68 ± 1.02	3 (1)	2.39 ± 0.95	2 (1)	0.0001
I am satisfied with the quality of gluten-free products available in the store/market	2.91 ± 1.07	3 (2)	2.90 ± 0.96	3 (2)	0.9372
I believe that the gluten-free products available in the store/supermarket are as good as their gluten-containing counterparts	2.59 ± 1.22	2 (1)	2.76 ± 1.12	3 (2)	0.0264
I like gluten-free products that I buy in a store/supermarket	3.28 ± 1.04	3 (1)	3.22 ± 0.90	3 (1)	0.2951
I trust the information on the labels of products marked as gluten-free in a store/market	3.73 ± 1.09	4 (2)	3.61 ± 1.10	4 (1)	0.1482
I prefer to make my own gluten-free products whenever possible	3.54 ± 1.35	4 (3)	3.76 ± 1.22	4 (2)	0.0444
I believe that when I buy gluten-free products in a store/supermarket, I get good quality at a reasonable price	2.39 ± 1.07	2 (1)	2.40 ± 1.00	2 (1)	0.7032
I often have to go to several different stores to get the gluten-free products I need	4.54 ± 0.90	5 (1)	4.66 ± 0.83	5 (0)	0.0097
Sometimes I have to go to another city to buy the gluten-free products I need	2.82 ± 1.70	3 (4)	3.10 ± 1.77	3 (4)	0.0259
I think the shelves in the “free” section of the store/supermarket are well-stocked	2.25 ± 0.99	2 (2)	2.15 ± 0.94	2 (2)	0.1637

* scale from 1 = strongly disagree to 5 = strongly agree; ** nonparametric distribution for all statements (Shapiro–Wilk test; *p* ≤ 0.05); SD—standard deviation; IQR—interquartile range.

**Table 5:** Opinions about gluten-free products’ availability and satisfaction in the studied group of Polish female coeliac-disease patients and female family members/relatives of patients diagnosed with coeliac disease, stratified by primary place of purchasing major grocery shopping.

Statements about Gluten-Free Products Available in Stores/Markets	Declared Agreement with the Statements * (*n* = 819)				*p*
	Primary Grocery Shopping in Hypermarkets (*n* = 595)		Primary Grocery Shopping Not in Hypermarkets (*n* = 224)		
	Mean ± SD	Median (IQR) **	Mean ± SD	Median (IQR) **	
I am satisfied with the range of gluten-free products available in the store/market	2.63 ± 1.00	3 (1)	2.45 ± 1.02	2 (1)	0.0150
I am satisfied with the quality of gluten-free products available in the store/market	2.95 ± 1.06	3 (2)	2.79 ± 0.95	3 (1)	0.0223
I believe that the gluten-free products available in the store/supermarket are as good as their gluten-containing counterparts	2.65 ± 1.20	3 (2)	2.63 ± 1.15	3 (1)	0.8516
I like gluten-free products that I buy in a store/supermarket	3.28 ± 0.98	3 (1)	3.20 ± 1.02	3 (1)	0.3038
I trust the information on the labels of products marked as gluten-free in a store/market	3.68 ± 1.10	4 (2)	3.72 ± 1.08	4 (2)	0.6981
I prefer to make my own gluten-free products whenever possible	3.61 ± 1.32	4 (2)	3.63 ± 1.29	4 (2)	0.8748
I believe that when I buy gluten-free products in a store/supermarket, I get good quality at a reasonable price	2.40 ± 1.03	2 (1)	2.39 ± 1.10	2 (2)	0.8551
I often have to go to several different stores to get the gluten-free products I need	4.58 ± 0.86	5 (1)	4.57 ± 0.94	5 (0)	0.6279
Sometimes I have to go to another city to buy the gluten-free products I need	2.82 ± 1.73	2 (4)	3.16 ± 1.71	3.5 (4)	0.0114
I think the shelves in the “free” section of the store/supermarket are well-stocked	2.25 ± 0.98	2 (2)	2.14 ± 0.96	2 (2)	0.1670

* scale from 1 = strongly disagree to 5 = strongly agree; ** nonparametric distribution for all statements (Shapiro–Wilk test; *p* ≤ 0.05); SD—standard deviation; IQR—interquartile range.

**Table 6:** Opinions about gluten-free products’ availability and satisfaction in the studied group of Polish female coeliac-disease patients and female family members/relatives of patients diagnosed with coeliac disease, stratified by whether gluten-free products were at least occasionally bought online.

Statements about Gluten-Free Products Available in Stores/Markets	Declared Agreement with the Statements * (*n* = 819)				*p*
	Gluten-Free Products at Least Occasionally Bought Online (*n* = 699)		Gluten-Free Products Not Bought Online (*n* = 120)		
	Mean ± SD	Median (IQR) **	Mean ± SD	Median (IQR) **	
I am satisfied with the range of gluten-free products available in the store/market	2.55 ± 1.01	3 (1)	2.77 ± 0.99	3 (1)	0.0253
I am satisfied with the quality of gluten-free products available in the store/market	2.88 ± 1.04	3 (2)	3.09 ± 0.98	3 (2)	0.0316
I believe that the gluten-free products available in the store/supermarket are as good as their gluten-containing counterparts	2.63 ± 1.18	3 (1)	2.77 ± 1.23	3 (2)	0.3033
I like gluten-free products that I buy in a store/supermarket	3.23 ± 0.98	3 (1)	3.41 ± 1.08	3 (1)	0.0786
I trust the information on the labels of products marked as gluten-free in a store/market	3.69 ± 1.10	4 (2)	3.73 ± 1.07	4 (2)	0.6842
I prefer to make my own gluten-free products whenever possible	3.66 ± 1.28	4 (2)	3.36 ± 1.44	3.5 (3)	0.0430
I believe that when I buy gluten-free products in a store/supermarket, I get good quality at a reasonable price	2.38 ± 1.05	2 (1)	2.49 ± 1.02	2 (1)	0.2317
I often have to go to several different stores to get the gluten-free products I need	4.61 ± 0.86	5 (0)	4.43 ± 0.98	5 (1)	0.0082
Sometimes I have to go to another city to buy the gluten-free products I need	3.01 ± 1.73	3 (4)	2.34 ± 1.62	1 (3)	0.0001
I think the shelves in the “free” section of the store/supermarket are well-stocked	2.19 ± 0.97	2 (2)	2.37 ± 0.99	2 (1)	0.0741

* scale from 1 = strongly disagree to 5 = strongly agree; ** nonparametric distribution for all statements (Shapiro–Wilk test; *p* ≤ 0.05); SD—standard deviation; IQR—interquartile range.

**Table 7:** Opinions about gluten-free products’ availability and satisfaction in the studied group of Polish female coeliac-disease patients and female family members/relatives of patients diagnosed with coeliac disease, stratified by declared problem with the availability of gluten-free products.

Statements about Gluten-Free Products Available in Stores/Markets	Declared Agreement with the Statements * (*n* = 819).				*p*
	Declared Problem with the Availability of Gluten-Free Products (*n* = 795)		No Declared Problem with the Availability of Gluten-Free Products (*n* = 24)		
	Mean ± SD	Median (IQR) **	Mean ± SD	Median (IQR) **	
I am satisfied with the range of gluten-free products available in the store/market	2.56 ± 1.00	3 (1)	3.33 ± 0.96	3 (1)	0.0008
I am satisfied with the quality of gluten-free products available in the store/market	2.90 ± 1.04	3 (2)	3.25 ± 0.94	3 (1)	0.1365
I believe that the gluten-free products available in the store/supermarket are as good as their gluten-containing counterparts	2.63 ± 1.18	3 (1)	3.25 ± 1.29	3.5 (2)	0.0245
I like gluten-free products that I buy in a store/supermarket	3.24 ± 0.99	3 (1)	3.79 ± 1.06	4 (2)	0.0093
I trust the information on the labels of products marked as gluten-free in a store/market	3.68 ± 1.10	4 (2)	4.00 ± 1.02	4 (1.5)	0.1532
I prefer to make my own gluten-free products whenever possible	3.62 ± 1.31	4 (2)	3.54 ± 1.28	4 (2)	0.7173
I believe that when I buy gluten-free products in a store/supermarket, I get good quality at a reasonable price	2.39 ± 1.05	2 (1)	2.58 ± 1.10	2.5 (1)	0.4034
I often have to go to several different stores to get the gluten-free products I need	4.60 ± 0.85	5 (0)	3.79 ± 1.44	4 (2)	0.0001
Sometimes I have to go to another city to buy the gluten-free products I need	2.94 ± 1.72	3 (4)	1.88 ± 1.60	1 (1)	0.0017
I think the shelves in the “free” section of the store/supermarket are well-stocked	2.19 ± 0.96	2 (2)	3.00 ± 1.14	3 (1.5)	0.0009

* scale from 1 = strongly disagree to 5 = strongly agree; ** nonparametric distribution for all statements (Shapiro–Wilk test; *p* ≤ 0.05); SD—standard deviation; IQR—interquartile range.

**Table 8:** Opinions about gluten-free products’ availability and satisfaction in the studied group of Polish female coeliac-disease patients and female family members/relatives of patients diagnosed with coeliac disease, stratified by declared problem with the quality of gluten-free products.

Statements about Gluten-Free Products Available in Stores/Markets	Declared Agreement with the Statements * (*n* = 819)				*p*
	Declared Problem with the Quality of Gluten-Free Products (*n* = 509)		No Declared Problem with the Quality of Gluten-Free Products (*n* = 310)		
	Mean ± SD	Median (IQR) **	Mean ± SD	Median (IQR) **	
I am satisfied with the range of gluten-free products available in the store/market	2.52 ± 0.98	3 (1)	2.68 ± 1.04	3 (1)	0.0282
I am satisfied with the quality of gluten-free products available in the store/market	2.7 ± 1.00	3 (1)	3.25 ± 1.01	3 (1)	<0.0001
I believe that the gluten-free products available in the store/supermarket are as good as their gluten-containing counterparts	2.43 ± 1.15	2 (2)	3.01 ± 1.16	3 (2)	<0.0001
I like gluten-free products that I buy in a store/supermarket	3.10 ± 0.98	3 (2)	3.51 ± 0.97	4 (1)	<0.0001
I trust the information on the labels of products marked as gluten-free in a store/market	3.61 ± 1.14	4 (2)	3.82 ± 1.00	4 (2)	0.0189
I prefer to make my own gluten-free products whenever possible	3.68 ± 1.29	4 (2)	3.52 ± 1.35	4 (3)	0.1139
I believe that when I buy gluten-free products in a store/supermarket, I get good quality at a reasonable price	2.23 ± 1.02	2 (2)	2.67 ± 1.04	3 (1)	<0.0001
I often have to go to several different stores to get the gluten-free products I need	4.62 ± 0.84	5 (0)	4.52 ± 0.94	5 (1)	0.1225
Sometimes I have to go to another city to buy the gluten-free products I need	2.95 ± 1.72	3 (4)	2.85 ± 1.74	3 (4)	0.4467
I think the shelves in the “free” section of the store/supermarket are well-stocked	2.11 ± 0.97	2 (2)	2.39 ± 0.95	2 (1)	<0.0001

* scale from 1 = strongly disagree to 5 = strongly agree; ** nonparametric distribution for all statements (Shapiro–Wilk test; *p* ≤ 0.05); SD—standard deviation; IQR—interquartile range.

## Data Availability

Data provided on request.

## References

[1] Posner E.B., Haseeb M. (2023). Celiac Disease.

[2] Tarar Z.I., Zafar M.U., Farooq U., Basar O., Tahan V., Daglilar E. (2021). The Progression of Celiac Disease, Diagnostic Modalities, and Treatment Options. J. Investig. Med. High Impact Case Rep..

[3] Singh P., Arora A., Strand T.A., Leffler D.A., Catassi C., Green P.H., Kelly C.P., Ahuja V., Makharia G.K. (2018). Global Prevalence of Celiac Disease: Systematic Review and Meta-analysis. Clin. Gastroenterol. Hepatol..

[4] Parzanese I., Qehajaj D., Patrinicola F., Aralica M., Chiriva-Internati M., Stifter S., Elli L., Grizzi F. (2017). Celiac disease: From pathophysiology to treatment. World J. Gastrointest. Pathophysiol..

[5] Caio G., Volta U., Sapone A., Leffler D.A., De Giorgio R., Catassi C., Fasano A. (2019). Celiac disease: A comprehensive current review. BMC Med..

[6] Ghazanfar H., Javed N., Lee S., Shaban M., Cordero D., Acherjee T., Hasan K.Z., Jyala A., Kandhi S., Hussain A.N. (2023). Novel Therapies for Celiac Disease: A Clinical Review Article. Cureus.

[7] Ludvigsson J.F., Bai J.C., Biagi F., Card T.R., Ciacci C., Ciclitira P.J., Green P.H., Hadjivassiliou M., Holdoway A., van Heel D.A. (2014). Diagnosis and management of adult coeliac disease: Guidelines from the British Society of Gastroenterology. Gut.

[8] Makovicky P., Makovicky P., Lupan I., Samasca G., Sur G., Freeman H.J. (2017). Perspective: Gluten-Free Products for Patients with Celiac Disease Should Not Contain Trace Levels. Adv. Nutr..

[9] Commission Implementing Regulation (EU) No 828/2014 of 30 July 2014 on the Requirements for the Provision of Information to Consumers on the Absence or Reduced Presence of Gluten in Food Text with EEA Relevance. https://eur-lex.europa.eu/legal-content/EN/TXT/?uri=CELEX%3A32014R0828.

[10] Hall N.J., Rubin G., Charnock A. (2009). Systematic review: Adherence to a gluten-free diet in adult patients with coeliac disease. Aliment. Pharmacol. Ther..

[11] Aspasia S., Emmanuela-Kalliopi K., Nikolaos T., Eirini S., Ioannis S., Anastasia M. (2022). The gluten-free diet challenge in adults with coeliac disease: The Hellenic survey. PEC Innov..

[12] Aljada B., Zohni A., El-Matary W. (2021). The Gluten-Free Diet for Celiac Disease and Beyond. Nutrients.

[13] Lee A.R., Wolf R.L., Lebwohl B., Ciaccio E.J., Green P.H.R. (2019). Persistent Economic Burden of the Gluten Free Diet. Nutrients.

[14] Hanci O., Jeanes Y.M. (2019). Are gluten-free food staples accessible to all patients with coeliac disease?. Frontline Gastroenterol..

[15] Zysk W., Głąbska D., Guzek D. (2018). Social and Emotional Fears and Worries Influencing the Quality of Life of Female Celiac Disease Patients Following a Gluten-Free Diet. Nutrients.

[16] Myhrstad M.C.W., Slydahl M., Hellmann M., Garnweidner-Holme L., Lundin K.E.A., Henriksen C., Telle-Hansen V.H. (2021). Nutritional quality and costs of gluten-free products: A case-control study of food products on the Norwegian marked. Food Nutr. Res..

[17] Zysk W., Głąbska D., Guzek D. (2019). Role of Front-of-Package Gluten-Free Product Labeling in a Pair-Matched Study in Women with and without Celiac Disease on a Gluten-Free Diet. Nutrients.

[18] Zysk W., Głąbska D., Guzek D. (2019). Food Neophobia in Celiac Disease and Other Gluten-Free Diet Individuals. Nutrients.

[19] Mącik R., Mącik D., Nalewajek M. Consumer preferences for retail format choice—Case of polish consumers. Proceedings of the Active Citizenship by Knowledge Management & Innovation Proceedings of the Management, Knowledge and Learning International Conference.

[20] Pechey R., Monsivais P. (2015). Supermarket Choice, Shopping Behavior, Socioeconomic Status, and Food Purchases. Am. J. Prev. Med..

[21] King J.A., Jeong J., Underwood F.E., Quan J., Panaccione N., Windsor J.W., Coward S., DeBruyn J., Ronksley P.E., Shaheen A.A. (2020). Incidence of Celiac Disease Is Increasing over Time: A Systematic Review and Meta-analysis. Am. J. Gastroenterol..

[22] Pinkhasov R.M., Wong J., Kashanian J., Lee M., Samadi D.B., Pinkhasov M.M., Shabsigh R. (2010). Are men shortchanged on health? Perspective on health care utilization and health risk behavior in men and women in the United States. Int. J. Clin. Pract..

[23] de Koning W., Botero R.J., Harnett J.E., Cunich M., Okati L., Mc Namara A., Vriesekoop F. (2024). Price, quality, and availability of gluten-free products in Australia and New Zealand—A cross-sectional study. J. Roy. Soc. N. Z..

[24] Bouasla A., Saouchi S., Benramoul K., Vriesekoop F. (2024). Availability and cost of gluten-free products in Algeria. Br. Food J..

[25] Majsiak E., Choina M., Golicki D., Gray A.M., Cukrowska B. (2021). The impact of symptoms on quality of life before and after diagnosis of coeliac disease: The results from a Polish population survey and comparison with the results from the United Kingdom. BMC Gastroenterol..

[26] Østerås N., Gulbrandsen P., Garratt A., Benth J.S., Dahl F.A., Natvig B., Brage S. (2008). A randomised comparison of a four- and a five-point scale version of the Norwegian Function Assessment Scale. Health Qual. Life Outcomes.

[27] Garratt A.M., Helgeland J., Gulbrandsen P. (2011). Five-point scales outperform 10-point scales in a randomized comparison of item scaling for the Patient Experiences Questionnaire. J. Clin. Epidemiol..

[28] World Health Organization (WHO) Process of Translation and Adaptation of Instruments. https://www.coursehero.com/file/30372721/WHO-Process-of-translation-and-adaptation-of-instrumentspdf/.

[29] Sperber A.D. (2004). Translation and validation of study instruments for cross-cultural research. Gastroenterology.

[30] Medley M.L. (1980). Life satisfaction across four stages of adult life. Int. J. Aging Hum. Dev..

[31] Central Statistical Office Statistical Office. https://stat.gov.pl/files/gfx/portalinformacyjny/pl/defaultaktualnosci/5499/3/7/1/miasta_w_liczbach_2012_07_31_internet.pdf.

[32] Majewska A., Denis M., Krupowicz W. (2020). Urbanization Chaos of Suburban Small Cities in Poland: ‘Tetris Development’. Land.

[33] Kutahyalioglu N.S., Kaş Alay G. (2024). Lived Experiences of Parents of Children with Celiac Disease: A Descriptive Qualitative Study. Pediatr. Gastroenterol. Hepatol. Nutr..

[34] Terms Used in Public Statistics. https://stat.gov.pl/metainformacje/slownik-pojec/pojecia-stosowane-w-statystyce-publicznej/1003,pojecie.html.

[35] Retail Market in Poland. https://www.wiadomoscihandlowe.pl/raporty-i-analizy-rynkowe/rynek-handlu-detalicznego-w-polsce-2429827.

[36] Hassan H.F., Mourad L., Khatib N., Assi R., Akil S., Khatib S.E., Hteit R. (2024). Perceptions towards gluten free products among consumers: A narrative review. Appl. Food Res..

[37] Alencar N.M.M., de Araújo V.A., Faggian L., da Silveira Araújo M.B., Capriles V.D. (2021). What about gluten-free products? An insight on celiac consumers’ opinions and expectations. J. Sens. Stud..

[38] Pohoreski K., Horwitz S.L., Gidrewicz D. (2023). Gluten-Free Diet Knowledge and Adherence in Adolescents with Celiac Disease: A Cross-Sectional Study. JPGN Rep..

[39] Marsilio I., Savarino E.V., Barberio B., Lorenzon G., Maniero D., Cingolani L., D’Odorico A., D’Incà R., Zingone F. (2020). A Survey on Nutritional Knowledge in Coeliac Disease Compared to Inflammatory Bowel Diseases Patients and Healthy Subjects. Nutrients.

[40] Masih J., Sharma A., Sharma A., Deutsch J. (2017). Study on Gap Estimation between Market Potential and Marketshare of Gluten-Free Market. Int. J. Curr. Microbiol. Appl. Sci..

[41] Lis D.M., Stellingwerff T., Shing C.M., Ahuja K.D., Fell J.W. (2015). Exploring the popularity, experiences, and beliefs surrounding gluten-free diets in nonceliac athletes. Int. J. Sport. Nutr. Exerc. Metab..

[42] Sangiorgio P., Errico S., Verardi A., Massa S., Pagliarello R., Marusic C., Lico C., Presenti O., Donini M., Baschieri S. (2023). Consumer Awareness and Acceptance of Biotechnological Solutions for Gluten-Free Products. Foods.

[43] Reilly N.R. (2016). The Gluten-Free Diet: Recognizing Fact, Fiction, and Fad. J. Pediatr..

[44] Niland B., Cash B.D. (2018). Health Benefits and Adverse Effects of a Gluten-Free Diet in Non-Celiac Disease Patients. Gastroenterol. Hepatol..

[45] Missbach B., Schwingshackl L., Billmann A., Mystek A., Hickelsberger M., Bauer G., König J. (2015). Gluten-free food database: The nutritional quality and cost of packaged gluten-free foods. Peer J..

[46] Lerner A., O’Bryan T., Matthias T. (2019). Navigating the Gluten-Free Boom: The Dark Side of Gluten Free Diet. Front. Pediatr..

[47] Kutlu T. (2019). Gluten-free diet: Is it really always beneficial?. Turk. Pediatri. Ars..

[48] Watson E. Health/Weight-Conscious Consumers Are Driving the Gluten-Free Market, Not Celiacs. https://www.foodnavigator-usa.com/Article/2013/10/15/Healthy-eaters-dieters-not-celiacs-propelling-gluten-free-market#.

[49] Global Gluten-Free Food Market Value from 2022 to 2032 (in Billion U.S. Dollars). https://www.statista.com/statistics/248467/global-gluten-free-food-market-size/.

[50] Burden M., Mooney P.D., Blanshard R.J., White W.L., Cambray-Deakin D.R., Sanders D.S. (2015). Cost and availability of gluten-free food in the UK: In store and online. Postgrad. Med. J..

[51] Posterick A., Ayars C.L. (2023). Celiac Disease Dietary Adherence on the Rural-Urban Continuum. Nutrients.

[52] Monzani A., Lionetti E., Felici E., Fransos L., Azzolina D., Rabbone I., Catassi C. (2020). Adherence to the Gluten-Free Diet during the Lockdown for COVID-19 Pandemic: A Web-Based Survey of Italian Subjects with Celiac Disease. Nutrients.

[53] Park J., Hill W.T. (2018). Exploring the role of justification and cognitive effort exertion on post-purchase regret in online shopping. Comput. Human Behav..

[54] Miller Y.D., Brown W.J. (2005). Determinants of Active Leisure for Women with Young Children—An “Ethic of Care” Prevails. Leisure Sci..

[55] Gilligan C. (1993). In a Different Voice: Psychological Theory and Women’s Development.

[56] Moore J., Abetz J.S. (2019). What Do Parents Regret About Having Children? Communicating Regrets Online. J. Fam. Issues.

[57] Baker K., Claridge A.M. (2022). “I have a Ph.D. in my daughter”: Mother and Child Experiences of Living with Childhood Chronic Illness. J. Child. Fam. Stud..

